# The New Nursing Standard for Rate-Supported Institutions

**Published:** 1920-03-13

**Authors:** 


### THE MATRONS' AND SISTERS' DEPARTMENT.

### THE NEW NURSING STANDARD FOR RATE-SUPPORTED INSTITUTIONS.

It was always anticipated that with the coming of the Health Ministry a new era was about to dawn for the nurses in Poor-law institutions. So far as concerns many of the large self-dependent infirmaries with training-schools for nurses, an upward trend has been observable for many years, and we have often had occasion to note the extent to which their standard of nursing approximates to that of the voluntary hospitals. But even to this there are exceptions, while the conditions in far too many of the smaller Poor-law institutions continue deplorable. The hour has now struck for reform, and the Health Minister has begun to make known the principles to be observed in nursing the sick and chronically infirm throughout the country.

At a recent meeting of the Medway Board of Guardians it was reported that Mr. Snowden, the General Inspector, and Dr. Fuller, the Medical Inspector, of the Health Ministry had attended the Nursing and Hospital Committee meeting and had explained the requirements of the Ministry of Health. These requirements relate, first, to the medical and surgical staff in institutions with a resident medical officer, and provide for the appointment in all institutions containing 200 beds and over of an assistant resident medical officer, a visiting medical officer, a visiting surgical officer, and a visiting children's diseases officer. As regards the nursing staff every effort is to be made to bring it into a state of greater efficiency. To this end it is laid down that the proportion of patients to nurses should not be more than six to one, both in the case of day and night nurses; that in infirm wards the proportion should not be more than nine to one; that every institution should train as many probationers as they could, giving them so far as possible both surgical and midwifery training; that the nurses should work under an eight-hour system; and that board wages should be allowed to nurses when on holiday.

When this charter comes into force throughout the country—at present it is put forth tentatively—it cannot fail completely to revolutionise the system under which the sick poor are ministered to. The expansion of the medical staff is a reform urgently called for in the interests of the sick, and will react very favourably indeed in the training of nurses. The introduction of the visiting physician and surgeon into the Poor-law infirmary will, it may confidently be anticipated, bring in its train a new spirit of interest and zeal hitherto sadly lacking. The overburdened medical officers of Poor-law institutions have given liberally of their scanty leisure to help in the task of training probationers, but every one knows how hard it has been in many places for nurses to get the opportunities they ought to have before calling themselves trained. The matron may do what she will, she cannot evolve skilled nurses unless she be seconded by a keen medical staff, with time for giving attention to individuals.

The new rule reducing the average number of patients in proportion to each nurse to six may not be considered very magnificent by some. It is true that in provincial hospitals the average works out at from three to five, and in most London hospitals does not exceed two or one and a-half. But the observance of the rule of six beds to a nurse would actually halve the work in many Poor-law establishments during the day and introduce an entirely new system by night. It is in the night that inmates of the rate-supported institutions have too long been exposed to scanty and hurried attention from harried nurses, if they have not been altogether neglected. The reform of night nursing will prove one of the greatest boons the Health Ministry has to bestow on nurses and patients alike.

We rejoice that the word has gone forth for setting an eight-hour day in these establishments. There is no mention of one day's rest in seven, so we are to conclude that it implies a fifty-six-hours week, but even this must lead to a considerable curtailment of duty hours in certain directions, and in institutions where the other officers enjoy a weekly day's rest it will not be possible long to exclude the nursing staff from its benefits. The provision of an allowance for board during the holidays will be more than commonly welcome at the present time.

There are many infirmaries where nurses could be trained in midwifery if the necessary equipment were furnished. Such re-organisation pre-supposes the removal of the unhappy Poor-law stigma from all rate-supported hospitals. Otherwise we could not reflect with satisfaction on the prospect. Mothers more and more readily seek institutional aid in child-birth, and well-equipped maternity departments in every infirmary would afford a great public benefit. More midwives, more trained nurses are the urgent necessity of the day. The new "suggestions" of the Health Ministry when loyally followed up will bring us both.

